# Safety assessment of tolvaptan: real-world adverse event analysis using the FAERS database

**DOI:** 10.3389/fphar.2024.1509310

**Published:** 2025-01-09

**Authors:** Peiyang Cao, Qian Wang, Yan Wang, Qing Qiao, Liyuan Yan

**Affiliations:** ^1^ Department of Nephrology, The Fourth Affiliated Hospital of Soochow University, Suzhou, Jiangsu, China; ^2^ Department of Cardiology, Affiliated Changshu Hospital of Nantong University, Changshu, China

**Keywords:** FAERS database, tolvaptan, adverse drug events, real-world data analysis, pharmacovigilance

## Abstract

**Objective:**

This study aims to analyze the adverse drug events (ADEs) associated with tolvaptan in the Food and Drug Administration Adverse Event Reporting System database from the fourth quarter of 2009 to the second quarter of 2024.

**Methods:**

After standardizing the data, various signal detection techniques, including Reporting Odds Ratio (ROR), Proportional Reporting Ratio (PRR), Bayesian Confidence Propagation Neural Network, and Multi-Item Gamma Poisson Shrinker, were employed for analysis.

**Results:**

Among the 7,486 ADE reports where tolvaptan was the primary suspected drug, a total of 196 preferred terms were identified, spanning 24 different system organ classes. Specifically, hepatobiliary disorders, renal and urinary disorders, and metabolic and nutritional disorders were found to be characteristic adverse reactions associated with tolvaptan. Additionally, uncommon but notable ADE signals were observed, such as renal cyst rupture, renal cyst infection, polycystic liver disease, and renal cyst hemorrhage. These several ADEs have not been referred to in the previous literature. Notably, strong ADE signals were detected for decreased urine osmolality [n = 5, ROR 149.74, PRR 149.7, IC (Information Component) 7.13, EBGM (Empirical Bayes Geometric Mean) 139.79], osmotic demyelination syndrome (n = 38, ROR 128.47, PRR 128.25, IC 6.92, EBGM 120.91), and pulmonary-related tumors such as bronchial metastatic carcinoma, bronchial carcinoma, metastatic small cell lung carcinoma, and small cell lung carcinoma. In the concomitant medication analysis of 7,486 suspected adverse drug reaction reports related to tolvaptan, the top three drugs most commonly used in combination with tolvaptan were furosemide, spironolactone, and amlodipine.

**Conclusion:**

While tolvaptan provides therapeutic benefits, it poses a risk of significant adverse reactions. Clinicians should closely monitor the occurrence of events related to hepatobiliary disorders, renal and urinary disorders, metabolic and nutritional disorders, as well as benign, malignant, and indeterminate tumors during its clinical use.

## 1 Introduction

Tolvaptan is a novel non-peptide selective vasopressin V2 receptor antagonist that works by inhibiting the binding of vasopressin to the V2 receptors in the renal collecting ducts. This action suppresses water reabsorption in the collecting ducts, promoting diuresis by increasing the excretion of electrolyte-free water without causing significant electrolyte loss (Kiuchi and Ikeda, 2019). Numerous studies have demonstrated that tolvaptan can reduce volume overload, stabilize hemodynamics, and improve hyponatremia without adversely affecting renal function (Kin et al., 2020; Tominaga et al., 2017). The U.S. Food and Drug Administration (FDA) has approved tolvaptan for the treatment of hypervolemic or euvolemic hyponatremia, including conditions such as heart failure, cirrhosis,and syndrome of inappropriate antidiuretic hormone secretion (SIADH). Tolvaptan can alleviate fluid retention and edema caused by heart failure by promoting water excretion and reducing excess body fluids. In some cases, it is used to manage symptoms related to heart failure. In September 2017, tolvaptan received approval for an additional indication in the management of fluid retention associated with heart failure. Furthermore, by inhibiting the action of vasopressin in the kidneys, tolvaptan reduces the growth and size of renal cysts, thereby slowing the progression of renal function deterioration. As a result, regulatory authorities in countries such as the United States and Japan have also approved tolvaptan for the treatment of autosomal dominant polycystic kidney disease (ADPKD) (Chebib et al., 2018; Yamazaki et al., 2021).

Although tolvaptan offers significant clinical benefits,its widespread use in clinical settings inevitably presents some adverse effects. Common side effects including thirst, dehydration, increased urine output, hypernatremia, and potential hepatotoxicity (Ebrahimi et al., 2024). In April 2013, the U.S. FDA issued a safety alert indicating that tolvaptan carries a risk of serious and potentially fatal liver injury. Patients with existing liver disease should not take the drug, and warnings were added to the label regarding hepatotoxicity and the risk of osmotic demyelination syndrome due to rapid correction of hyponatremia. Therefore, in the actual medication process, there may be adverse reaction signals or even serious adverse drug reactions (ADRs) that are not mentioned in the drug instructions. Identifying potential safety signals for tolvaptan through data mining algorithms is highly desirable. To date, few studies have explored the adverse reaction signals of tolvaptan. In a previous study based on the Food and Drug Administration Adverse Event Reporting System (FAERS) database, the focus was primarily on the occurrence of thromboembolic events, particularly pulmonary embolism, associated with tolvaptan (Aiello et al., 2021). A more recent study analyzed tolvaptan-related liver disease using different databases and found that the severity of liver disease varied with age. The researchers suggested that patients should be monitored for liver function according to their age to prevent potentially fatal outcomes (Uno et al., 2024). These studies have mainly focused on investigating specific severe complications associated with tolvaptan. FAERS provides a platform for collecting and adverse drug events (ADEs) related to drug use, serving as a crucial resource for evaluating drug safety and efficacy. This paper aims to leverage various signal detection techniques to analyze the FAERS database, quantify the data on tolvaptan, and assess it from different perspectives, providing more comprehensive and reliable results.

## 2 Methods

### 2.1 Data source

**FIGURE 1:**
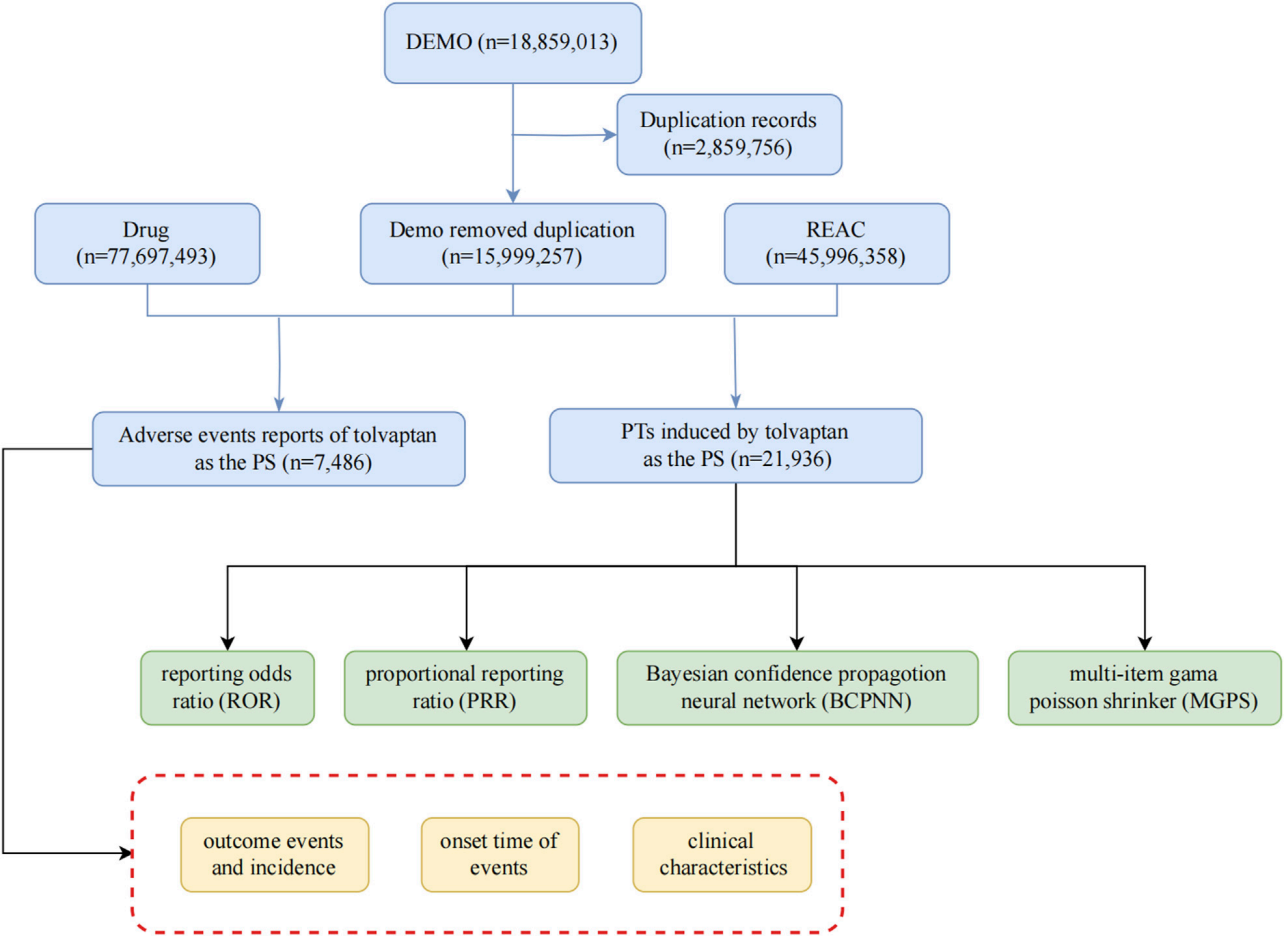
The flow diagram of selecting tolvaptan-related adverse events from FAERS database.

Given the drug’s approval date, this study utilized report files from the FAERS database, encompassing the period from the fourth quarter of 2009 to the second quarter of 2024, employing the American Standard Code for Information Interchange (ASCII). For this research, FAERS data were processed and analyzed using R software (version 4.4.1), which included data collection and cleaning. The methodology involved encoding and categorizing Preferred Terms (PTs) based on the Drug Adverse Event Terminology Set and system organ class (SOC), as defined in the Medical Dictionary for Regulatory Activities (MedDRA) (Brown, 2004), to evaluate the SOC categories implicated in adverse events (AEs). The research team collected data on relevant adverse reactions and identified all associated PTs corresponding to the SOC. Our investigation focused on examining AEs linked to tolvaptan, which was identified in the FAERS database as a primary suspect drug. The study assessed serious clinical outcomes, encompassing death, disability, hospitalization, and life-threatening situations. Additionally, the analysis took into account factors such as gender, age, and the reporting country. The comprehensive screening procedure was depicted in Figure 1.

### 2.2 Data extraction and identification

To identify relevant AE reports, we conducted a search using “tolvaptan” as the generic name, concentrating on cases where tolvaptan was designated as the primary suspect drug. To ensure data integrity and reliability, we adhered strictly to the FDA’s official guidance for data cleaning. Our deduplication process focused on the DEMO table’s PRIMARYID, CASEID, and FDA_DT fields. We sorted the dataset by CASEID, FDA_DT, and PRIMARYID to systematically identify duplicates. For reports with identical CASEID, we retained only the entry with the most recent FDA_DT, ensuring the most up-to-date information was preserved. In cases where both CASEID and FDA_DT were identical, the report with the highest PRIMARYID was retained, maintaining the most comprehensive version of each report. This rigorous approach, aligned with FDA recommendations, effectively eliminated redundant entries and ensured the robustness of our subsequent analyses. Medication nomenclature was standardized using the Medex_UIMA_1.8.3 system. The main function of the system is to automatically extract key information from medical literature, such as drug name, indication, dosage, etc. Data from different sources are integrated to facilitate subsequent analysis. Provide clinicians with accurate information to support drug selection and treatment options. The Medex_UIMA_1.8.3 system has unique advantages in the field of medical information processing, especially in the standardization of drug names, and through its flexible and efficient design, it can provide important support for medical institutions. Its accuracy and integration capabilities make it more effective in clinical applications compared to other systems. For the categorization of reported AEs, we adhered to the standardized coding of PTs as outlined in version 26.1 of MedDRA.

### 2.3 Statistical analysis

Our study employed multiple disproportionality analysis methods, including Reporting Odds Ratio (ROR), Proportional Reporting Ratio (PRR), Bayesian Confidence Propagation Neural Network (BCPNN), and Multi-item Gamma Poisson Shrinker (MGPS), each selected for their complementary strengths and specific applications to our dataset. The ROR was chosen as a primary method due to its established use in pharmacovigilance and effectiveness in large-scale spontaneous reporting databases (Rothman et al., 2004). The PRR was selected for its robust performance with smaller sample sizes and stability in analyzing rare AEs, aligning with FDA’s standard methodological approach (Evans et al., 2001). The BCPNN was implemented to address random errors in small samples and provide reliable signal strength estimates through Information Component (IC) (Bate et al., 1998), while the MGPS was particularly valuable for analyzing multiple drug-event combinations and reducing false positives through Bayesian shrinkage (Dumouchel, 1999). To address potential confounding factors and bias, we conducted stratified analyses based on age, gender, and weight. All of these algorithms are founded on a 2 × 2 contingency table (Table 1). The specific formulas and corresponding thresholds are presented in Table 2. Our signal detection thresholds were meticulously established through comprehensive review of literature and rigorous empirical validation. For disproportionality analysis, we implemented several well-validated criteria across different methods. The PRR analysis employed thresholds of PRR ≥2 and N ≥ 3, adhering to European Medicines Agency guidelines which have demonstrated optimal balance between sensitivity and specificity in pharmacovigilance studies (Evans et al., 2001). For ROR analysis, we adopted more stringent criteria with ROR ≥ 3 and the lower bound of the 95% confidence interval (CI) > 1, specifically chosen to enhance signal specificity and reduce false positives in our context (van Puijenbroek et al., 2002). The BCPNN method utilized IC025 (Information Component 2.5th percentile) > 0, a threshold established to effectively minimize false positives while preserving adequate signal detection sensitivity (Bate et al., 1998). For MGPS analysis, we implemented EB05 (Empirical Bayes 5th percentile) > 2, a conservative threshold that effectively controls for multiple comparisons in large-scale signal detection (Szarfman et al., 2002). The robustness of these thresholds was extensively validated through multiple approaches, including comparisons with established AEs, comprehensive sensitivity analyses across different threshold values, and systematic performance evaluations using both positive and negative controls (Hauben et al., 2005). This thorough validation framework ensures both the reliability and methodological integrity of our signal detection process. All statistical analyses were performed using R software version 4.4.1.

**TABLE 1:** Fourfold table of disproportionality method.

	Target adverse events reported	Other adverse events reported	Total
Target drug	a	b	a + b
Other drugs	c	d	c + d
Total	a + c	b + d	a + b + c + d

**TABLE 2:** ROR, PRR, BCPNN, and EBGM methods, formulas, and thresholds.

Method	Formula	Threshold
ROR	$\text{ROR}=\frac{a\;/\;c}{b\;/\;d}$	a ≥3
	$SE\left(lnROR\right)=\sqrt{\frac{1}{a}+\frac{1}{b}+\frac{1}{c}+\frac{1}{d}}$	ROR ≥3
	$95\%CI=e^{\ln\left(ROR\right)\pm 1.96se}$	95%CI (lower limit) > 1
PRR	$\text{PRR}=\frac{a\;/\;\left(a+b\right)}{c\;/\;\left(c+d\right)}$	a ≥3
	$SE\left(lnPRR\right)=\sqrt{\frac{1}{a}-\frac{1}{a+b}+\frac{1}{c}-\frac{1}{c+d}}$	PRR ≥2
	$95\%CI=e^{\ln\left(PRR\right)\pm 1.96se}$	95%CI (lower limit) > 1
BCPNN	$\text{IC}=\log_{2}\frac{p\left(x,y\right)}{p\left(x\right)p\left(y\right)}=\log_{2}\frac{a\left(a+b+c+d\right)}{\left(a+b\right)\left(a+c\right)}$	IC025 > 0
	$E\left(\text{IC}\right)=\log_{2}\frac{\left(a+\gamma 11\right)\left(a+b+c+d+\alpha \right)\left(a+b+c+d+\beta \right)}{\left(a+b+c+d+\gamma \right)\left(a+b+\alpha 1\right)\left(a+c+\beta 1\right)}$	
	$V\left(\text{IC}\right)=\frac{1}{{\left(\ln2\right)}^{2}}\left[\frac{\left(a+b+c+d\right)-a+\gamma -\gamma 11}{\left(a+\gamma 11\right)\left(1+a+b+c+d+\gamma \right)}+\frac{\left(a+b+c+d\right)-\left(a+b\right)+a-\alpha 1}{\left(a+b+\alpha 1\right)\left(1+a+b+c+d+\alpha \right)}+\frac{\left(a+b+c+d+\alpha \right)-\left(a+c\right)+\beta -\beta 1}{\left(a+b+\beta 1\right)\left(1+a+b+c+d+\beta \right)}\right]$	
	$\gamma =\gamma 11\frac{\left(a+b+c+d+\alpha \right)\left(a+b+c+d+\beta \right)}{\left(a+b+\alpha 1\right)\left(a+c+\beta 1\right)}$	
	$\text{IC}-2\text{SD}=E\left(\text{IC}\right)-2\;\sqrt{V\left(\text{IC}\right)}$	
EBGM	$\text{EBGM}=\frac{a\left(a+b+c+d\right)}{\left(a+c\right)\left(a+b\right)}$	EBGM05 > 2
	$SE\left(lnEBGM\right)=\sqrt{\frac{1}{a}+\frac{1}{b}+\frac{1}{c}+\frac{1}{d}}$	
	$95\%CI=e^{\ln\left(EBGM\right)\pm 1.96se}$	

## 3 Results

### 3.1 Basic characteristics of tolvaptan-related ADEs

From 2009 to 2024, a total of 15,999,257 adverse event reports were collected from the FAERS database. Among them, 7,486 reports identified tolvaptan as the primary suspected drug in ADEs. In these reports, there was no significant difference in the ratio of men to women. Regarding age distribution, Patients over 45 years of age accounted for the majority of individual case safety reports (54.59%), excluding those with unknown age. Most of the reports (43.55%) were submitted by Physicians. The United States was the country with the highest number of reports (41.77%). In terms of clinical outcomes, the most frequent serious ADEs, apart from those with unspecified severity, were those leading to hospitalization (25.36%), followed by death (20.49%). Supplementary Figure S1 shows the top 10 PTs associated with hospitalization and death. The most frequent PT related to death is cardiac failure (144 occurrences), followed by product use in unapproved indications (118 occurrences), and hypernatremia (110 occurrences). The top three PTs related to hospitalization are: product use in unapproved indications (151 occurrences), wrong technique in product usage (147 occurrences), and dehydration (146 occurrences). On the temporal distribution of ADE, excluding cases with unknown drug administration duration, the highest proportion of adverse reactions occurred within 7 days, accounting for 19.62%.The incidence of AEs decreased over time. Between days 7 and 28, 563 AEs were reported (7.52%), and between days 28 and 60, 281 AEs were reported (3.75%). Notably, our data suggest that 12.82% of patients may still experience AEs after ≥60 days of tolvaptan treatment. These findings underscore the importance of continuous monitoring for potential AEs throughout the entire duration of tolvaptan therapy. For further details, see Table 3. Supplementary Table S1 shows the frequency distribution of tolvaptan dosing regimens, with the most common dose being 15 mg once daily, administered to 1,029 patients. The next most common dose was 7.5 mg once daily, administered to 822 patients.

**TABLE 3:** Summary of adverse drug events (ADEs) associated with tolvaptan reported in the FAERS database.

Variable	Total
Year	
2009	13 (0.17)
2010	42 (0.56)
2011	101 (1.35)
2012	362 (4.84)
2013	392 (5.24)
2014	273 (3.65)
2015	318 (4.25)
2016	507 (6.77)
2017	633 (8.46)
2018	736 (9.83)
2019	997 (13.32)
2020	566 (7.56)
2021	527 (7.04)
2022	615 (8.22)
2023	693 (9.26)
2024	711 (9.50)
Sex	
female	3,247 (43.37)
male	3,258 (43.52)
unknown	981 (13.10)
Age	
<18	43 (0.57)
18–45	788 (10.53)
45–65	1720 (22.98)
65–75	791 (10.57)
≥75	1,575 (21.04)
unknown	2,569 (34.32)
Weight	
<40 kg	78 (1.04)
40–60 kg	546 (7.29)
60–80 kg	585 (7.81)
80–100 kg	325 (4.34)
≥100 kg	147 (1.96)
unknown	5,805 (77.54)
Reporter	
Physician	3,260 (43.55)
Consumer	1,691 (22.59)
Pharmacist	1,663 (22.21)
Other health-professional	633 (8.46)
unknown	233 (3.11)
Registered Nurse	4 (0.05)
Lawyer	2 (0.03)
Reported countries	
United States	3,127 (41.77)
Japan	2,280 (30.46)
other	670 (8.95)
Philippines	307 (4.10)
United Kingdom	298 (3.98)
Canada	283 (3.78)
Germany	189 (2.52)
China	101 (1.35)
France	92 (1.23)
Korea, South	71 (0.95)
Spain	68 (0.91)
Outcomes	
other serious	3,898 (49.49)
hospitalization	1997 (25.36)
death	1,614 (20.49)
life threatening	230 (2.92)
disability	122 (1.55)
required intervention to Prevent Permanent Impairment/Damage	10 (0.13)
congenital anomaly	5 (0.06)
Onset time of adverse effects (days)	
<7	1,469 (19.62)
7–28	563 (7.52)
28–60	281 (3.75)
≥60	960 (12.82)
unknown	4,213 (56.28)

A single primaryid may correspond to multiple outcomes.

### 3.2 Signal detection for tolvaptan

In this study, analysis of adverse event reports involving tolvaptan revealed that adverse reactions related to the drug involved 24 different SOCs. Statistically, the SOCs that met all four signal detection criteria and were significantly associated with tolvaptan-related AEs were hepatobiliary disorders (n = 942, ROR 4.87, PRR 4.7, IC 2.23, Empirical Bayes Geometric Mean (EBGM) 4.7), renal and urinary disorders (n = 1788, ROR 4.49, PRR 4.2, IC 2.07, EBGM 4.2), metabolic and nutritional disorders (n = 1,678, ROR 3.7, PRR 3.49, IC 1.8, EBGM 3.49), and investigations (n = 3,737, ROR 3.14, PRR 2.78, IC 1.47, EBGM 2.78). Details can be found in Table 4.

**TABLE 4:** The signal strength of ADEs of tolvaptan at the SOC level in FAERS database.

System organ class (SOC) name	Cases reporting SOC	ROR (95% CI)	PRR (95% CI)	IC (IC 025)	EBGM (EBGM 05)
hepatobiliary disorders	942	4.87 (4.56, 5.2)	4.7 (4.43, 4.98)	2.23 (2.14)	4.7 (4.45)
renal and urinary disorders	1788	4.49 (4.28, 4.71)	4.2 (4.04, 4.37)	2.07 (2)	4.2 (4.03)
metabolism and nutrition disorders	1,678	3.7 (3.52, 3.89)	3.49 (3.36, 3.63)	1.8 (1.73)	3.49 (3.35)
investigations	3,737	3.14 (3.03, 3.26)	2.78 (2.73, 2.84)	1.47 (1.42)	2.78 (2.7)
cardiac disorders	963	1.74 (1.63, 1.86)	1.71 (1.61, 1.81)	0.77 (0.68)	1.71 (1.62)
injury, poisoning and procedural complications	3,070	1.44 (1.38, 1.49)	1.37 (1.32, 1.42)	0.46 (0.4)	1.37 (1.33)
endocrine disorders	50	0.86 (0.65, 1.13)	0.86 (0.65, 1.13)	−0.22 (−0.61)	0.86 (0.68)
vascular disorders	379	0.79 (0.71, 0.87)	0.79 (0.72, 0.87)	−0.34 (−0.49)	0.79 (0.73)
gastrointestinal disorders	1,393	0.7 (0.66, 0.74)	0.72 (0.68, 0.76)	−0.48 (−0.56)	0.72 (0.68)
general disorders and administration site conditions	2,991	0.7 (0.67, 0.72)	0.74 (0.71, 0.77)	−0.44 (−0.5)	0.74 (0.71)
nervous system disorders	1,356	0.69 (0.66, 0.73)	0.71 (0.67, 0.75)	−0.49 (−0.57)	0.71 (0.68)
infections and infestations	829	0.67 (0.62, 0.71)	0.68 (0.64, 0.72)	−0.56 (−0.66)	0.68 (0.64)
blood and lymphatic system disorders	228	0.6 (0.52, 0.68)	0.6 (0.52, 0.69)	−0.74 (−0.92)	0.6 (0.54)
respiratory, thoracic and mediastinal disorders	641	0.58 (0.54, 0.63)	0.59 (0.55, 0.64)	−0.76 (−0.87)	0.59 (0.55)
neoplasms benign, malignant and unspecified (incl cysts and polyps)	361	0.56 (0.51, 0.63)	0.57 (0.52, 0.63)	−0.81 (−0.96)	0.57 (0.52)
ear and labyrinth disorders	47	0.48 (0.36, 0.63)	0.48 (0.36, 0.64)	−1.07 (−1.48)	0.48 (0.38)
psychiatric disorders	525	0.4 (0.36, 0.43)	0.41 (0.38, 0.44)	−1.29 (−1.41)	0.41 (0.38)
congenital, familial and genetic disorders	25	0.36 (0.24, 0.53)	0.36 (0.24, 0.53)	−1.47 (−2.03)	0.36 (0.26)
eye disorders	137	0.29 (0.25, 0.35)	0.3 (0.25, 0.36)	−1.74 (−1.98)	0.3 (0.26)
musculoskeletal and connective tissue disorders	355	0.28 (0.25, 0.31)	0.29 (0.26, 0.32)	−1.79 (−1.94)	0.29 (0.27)
reproductive system and breast disorders	50	0.27 (0.2, 0.35)	0.27 (0.21, 0.36)	−1.91 (−2.3)	0.27 (0.21)
skin and subcutaneous tissue disorders	333	0.26 (0.23, 0.28)	0.27 (0.24, 0.3)	−1.91 (−2.06)	0.27 (0.24)
immune system disorders	45	0.17 (0.13, 0.23)	0.17 (0.13, 0.23)	−2.53 (−2.94)	0.17 (0.14)
pregnancy, puerperium and perinatal conditions	13	0.14 (0.08, 0.24)	0.14 (0.08, 0.24)	−2.86 (−3.61)	0.14 (0.09)

ROR, reporting odds ratio; CI, confidence interval; PRR, proportional reporting ratio; IC, information component; IC 025, the lower limit of 95% CI of the IC, EBGM, empirical Bayesian geometric mean; EBGM 05, the lower limit of 95% CI of EBGM.

At the PT Level, four algorithms were used to analyze adverse drug reactions and assess their compliance with various screening criteria, identifying 196 PTs. The results were ranked according to the most stringent EBGM algorithm, as shown in Tables 5, 6. The findings revealed high signal intensity for certain PTs, such as renal cyst rupture (n = 17, ROR 419.49, PRR 419.17, IC 8.45, EBGM 349.47), renal cyst infection (n = 18, ROR 359.58, PRR 359.29, IC 8.26, EBGM 306.86), and polycystic liver disease (n = 8, ROR 243.08, PRR 243, IC 7.77, EBGM 217.85). The most commonly reported PTs were thirst (n = 473, ROR 75.79, PRR 74.17, IC 6.16, EBGM 71.67), renal impairment (n = 416, ROR 13.47, PRR 13.23, IC 3.72, EBGM 13.15), product use in unapproved indication (n = 390, ROR 4.44, PRR 4.38, IC 2.13, EBGM 4.37),wrong technique in product usage process (n = 331, ROR 4.13, PRR 4.09, IC 2.03, EBGM 4.08), hypernatraemia (n = 326, ROR 199.8, PRR 196.84, IC 7.49, EBGM 180.03) and dehydration (n = 313, ROR 6.57, PRR 6.49, IC 2.7, EBGM 6.48). In addition to the side effects already mentioned in the drug label, this study identified renal cyst hemorrhage and decreased urine osmolality, which, although infrequent, exhibited high signal strength. Notably, pulmonary-related tumors such as bronchial metastatic carcinoma, bronchial carcinoma, metastatic small cell lung carcinoma, and small cell lung carcinoma were observed after tolvaptan treatment, with high signal intensity.

**TABLE 5:** The top 50 AEs of tolvaptan ranked by the frequency at the PTs level.

System organ class (SOC) name	Preferred terms (PTs)	Cases reporting PT	ROR (95% CI)	PRR (95% CI)	IC (IC025)	EBGM (EBGM05)
general disorders and administration site conditions	thirst	473	75.79 (69.08, 83.15)	74.17 (67.25, 81.81)	6.16 (6.03)	71.67 (66.33)
renal and urinary disorders	renal impairment	416	13.47 (12.22, 14.84)	13.23 (11.99, 14.59)	3.72 (3.58)	13.15 (12.13)
injury, poisoning and procedural complications	product use in unapproved indication	390	4.44 (4.01, 4.9)	4.38 (3.97, 4.83)	2.13 (1.98)	4.37 (4.02)
injury, poisoning and procedural complications	wrong technique in product usage process	331	4.13 (3.71, 4.61)	4.09 (3.71, 4.51)	2.03 (1.87)	4.08 (3.73)
metabolism and nutrition disorders	hypernatraemia	326	199.8 (178.21, 224)	196.84 (175, 221.4)	7.49 (7.33)	180.03 (163.61)
metabolism and nutrition disorders	dehydration	313	6.57 (5.88, 7.35)	6.49 (5.77, 7.3)	2.7 (2.53)	6.48 (5.9)
investigations	blood sodium decreased	309	50.43 (45.02, 56.5)	49.74 (44.22, 55.95)	5.6 (5.44)	48.61 (44.2)
renal and urinary disorders	polyuria	308	110.39 (98.37, 123.89)	108.86 (96.78, 122.45)	6.69 (6.53)	103.53 (94.01)
injury, poisoning and procedural complications	underdose	306	10.49 (9.37, 11.75)	10.36 (9.21, 11.65)	3.37 (3.2)	10.31 (9.38)
investigations	blood creatinine increased	300	13.22 (11.79, 14.82)	13.05 (11.6, 14.68)	3.7 (3.53)	12.98 (11.79)
cardiac disorders	cardiac failure	294	9.99 (8.9, 11.22)	9.87 (8.77, 11.1)	3.3 (3.13)	9.83 (8.93)
injury, poisoning and procedural complications	inappropriate schedule of product administration	290	4.76 (4.24, 5.35)	4.71 (4.19, 5.3)	2.23 (2.07)	4.7 (4.27)
investigations	blood urea increased	243	48.38 (42.57, 54.98)	47.85 (42.54, 53.82)	5.55 (5.36)	46.81 (42.06)
hepatobiliary disorders	hepatic function abnormal	233	18.51 (16.26, 21.07)	18.32 (15.97, 21.01)	4.18 (4)	18.17 (16.3)
renal and urinary disorders	nocturia	199	47.12 (40.92, 54.26)	46.7 (40.71, 53.57)	5.51 (5.31)	45.7 (40.61)
metabolism and nutrition disorders	hyponatraemia	198	9.85 (8.56, 11.33)	9.77 (8.52, 11.21)	3.28 (3.08)	9.73 (8.65)
investigations	alanine aminotransferase increased	196	10.06 (8.74, 11.58)	9.98 (8.7, 11.45)	3.31 (3.11)	9.93 (8.83)
investigations	glomerular filtration rate decreased	180	41.46 (35.75, 48.08)	41.13 (35.86, 47.18)	5.33 (5.12)	40.36 (35.65)
renal and urinary disorders	pollakiuria	163	10.65 (9.12, 12.43)	10.58 (9.04, 12.38)	3.4 (3.17)	10.53 (9.25)
investigations	aspartate aminotransferase increased	153	9.33 (7.96, 10.95)	9.27 (7.92, 10.84)	3.21 (2.98)	9.24 (8.09)
metabolism and nutrition disorders	polydipsia	148	116.91 (99.03, 138.03)	116.13 (99.28, 135.84)	6.78 (6.54)	110.09 (95.81)
injury, poisoning and procedural complications	incorrect product administration duration	146	18.53 (15.74, 21.82)	18.42 (15.75, 21.55)	4.19 (3.96)	18.26 (15.93)
investigations	blood sodium increased	137	162.77 (136.74, 193.77)	161.76 (135.6, 192.97)	7.23 (6.98)	150.24 (129.86)
hepatobiliary disorders	liver disorder	110	7.08 (5.86, 8.54)	7.04 (5.79, 8.56)	2.81 (2.54)	7.02 (6)
investigations	hepatic enzyme increased	105	4.35 (3.59, 5.27)	4.33 (3.56, 5.27)	2.11 (1.84)	4.32 (3.68)
nervous system disorders	hepatic encephalopathy	102	29.55 (24.29, 35.94)	29.41 (24.18, 35.78)	4.86 (4.58)	29.02 (24.63)
metabolism and nutrition disorders	hyperkalaemia	95	7.72 (6.31, 9.45)	7.69 (6.32, 9.36)	2.94 (2.65)	7.67 (6.48)
hepatobiliary disorders	liver injury	94	11.69 (9.54, 14.32)	11.64 (9.57, 14.16)	3.53 (3.24)	11.58 (9.77)
investigations	blood pressure decreased	75	3.18 (2.54, 3.99)	3.17 (2.51, 4.01)	1.66 (1.34)	3.17 (2.62)
nervous system disorders	altered state of consciousness	71	8.82 (6.98, 11.14)	8.8 (6.96, 11.13)	3.13 (2.8)	8.76 (7.21)
investigations	liver function test increased	67	8.39 (6.6, 10.67)	8.36 (6.61, 10.58)	3.06 (2.72)	8.34 (6.82)
hepatobiliary disorders	hepatic failure	66	6.42 (5.04, 8.18)	6.41 (5.07, 8.11)	2.68 (2.33)	6.39 (5.22)
metabolism and nutrition disorders	hypokalaemia	65	3.95 (3.1, 5.04)	3.94 (3.11, 4.98)	1.98 (1.63)	3.94 (3.21)
hepatobiliary disorders	hepatotoxicity	62	7.8 (6.08, 10.01)	7.78 (6.03, 10.04)	2.96 (2.6)	7.75 (6.29)
investigations	blood potassium increased	61	10.88 (8.46, 14)	10.85 (8.41, 14)	3.43 (3.07)	10.8 (8.75)
investigations	gamma-glutamyltransferase increased	56	7.91 (6.08, 10.29)	7.89 (6.12, 10.18)	2.98 (2.6)	7.87 (6.31)
investigations	blood alkaline phosphatase increased	56	7.29 (5.61, 9.48)	7.27 (5.63, 9.38)	2.86 (2.48)	7.25 (5.82)
investigations	transaminases increased	54	6.62 (5.07, 8.65)	6.61 (5.02, 8.7)	2.72 (2.34)	6.59 (5.27)
investigations	blood bilirubin increased	53	5.93 (4.52, 7.76)	5.91 (4.49, 7.78)	2.56 (2.17)	5.9 (4.71)
metabolism and nutrition disorders	hyperuricaemia	53	34.91 (26.61, 45.82)	34.83 (26.47, 45.83)	5.1 (4.71)	34.28 (27.31)
investigations	blood uric acid increased	48	23.31 (17.54, 31)	23.27 (17.69, 30.62)	4.52 (4.12)	23.02 (18.14)
investigations	blood creatine phosphokinase increased	48	5.21 (3.92, 6.92)	5.2 (3.95, 6.84)	2.38 (1.97)	5.19 (4.09)
investigations	blood potassium decreased	46	4.23 (3.17, 5.65)	4.22 (3.15, 5.66)	2.08 (1.66)	4.22 (3.31)
nervous system disorders	cerebral infarction	45	5.08 (3.79, 6.81)	5.07 (3.78, 6.8)	2.34 (1.92)	5.07 (3.96)
hepatobiliary disorders	hepatic cirrhosis	44	6.64 (4.94, 8.93)	6.63 (4.94, 8.9)	2.72 (2.3)	6.61 (5.16)
nervous system disorders	osmotic demyelination syndrome	38	128.47 (92.56, 178.3)	128.25 (91.91, 178.96)	6.92 (6.45)	120.91 (91.91)
investigations	urine output increased	38	37.56 (27.24, 51.78)	37.5 (27.41, 51.31)	5.2 (4.75)	36.85 (28.17)
investigations	liver function test abnormal	38	3.79 (2.75, 5.21)	3.78 (2.76, 5.17)	1.92 (1.46)	3.78 (2.89)
renal and urinary disorders	renal pain	38	9.66 (7.02, 13.29)	9.65 (7.05, 13.2)	3.26 (2.81)	9.61 (7.36)
injury, poisoning and procedural complications	prescribed underdose	38	4.72 (3.44, 6.5)	4.72 (3.45, 6.46)	2.24 (1.78)	4.71 (3.61)

ROR, reporting odds ratio; CI, confidence interval; PRR, proportional reporting ratio; IC, information component; IC 025, the lower limit of 95% CI of the IC; EBGM, empirical Bayesian geometric mean; EBGM 05: the lower limit of 95% CI of EBGM.

**TABLE 6:** The top 50 signal strength of AEs of tolvaptan ranked by the ROR at the PTs level.

System organ class (SOC)name	Preferred terms (PTs)	Cases reporting PT	ROR (95% CI)	PRR (95% CI)	IC (IC025)	EBGM (EBGM05)
renal and urinary disorders	renal cyst ruptured	17	419.49 (249.17, 706.24)	419.17 (246.92, 711.57)	8.45 (7.73)	349.47 (226.01)
Infections and infestations	renal cyst infection	18	359.58 (218.06, 592.96)	359.29 (215.84, 598.08)	8.26 (7.56)	306.86 (201.92)
congenital, familial and genetic disorders	polycystic liver disease	8	243.08 (116.89, 505.51)	243 (117.67, 501.83)	7.77 (6.77)	217.85 (118.06)
renal and urinary disorders	renal cyst haemorrhage	20	234.39 (147.62, 372.14)	234.17 (146.3, 374.82)	7.72 (7.07)	210.74 (143.14)
metabolism and nutrition disorders	hypernatraemia	326	199.8 (178.21, 224)	196.84 (175, 221.4)	7.49 (7.33)	180.03 (163.61)
investigations	blood sodium increased	137	162.77 (136.74, 193.77)	161.76 (135.6, 192.97)	7.23 (6.98)	150.24 (129.86)
investigations	urine osmolarity decreased	5	149.74 (60.43, 371.03)	149.7 (60.77, 368.79)	7.13 (5.93)	139.79 (65.42)
nervous system disorders	osmotic demyelination syndrome	38	128.47 (92.56, 178.3)	128.25 (91.91, 178.96)	6.92 (6.45)	120.91 (91.91)
metabolism and nutrition disorders	polydipsia	148	116.91 (99.03, 138.03)	116.13 (99.28, 135.84)	6.78 (6.54)	110.09 (95.81)
injury, poisoning and procedural complications	drug monitoring procedure incorrectly performed	28	116.12 (79.35, 169.93)	115.98 (79.92, 168.31)	6.78 (6.24)	109.95 (79.95)
renal and urinary disorders	polyuria	308	110.39 (98.37, 123.89)	108.86 (96.78, 122.45)	6.69 (6.53)	103.53 (94.01)
cardiac disorders	low cardiac output syndrome	13	105.67 (60.52, 184.49)	105.6 (61, 182.81)	6.65 (5.88)	100.59 (63.1)
infections and infestations	hepatic cyst infection	4	103.52 (37.93, 282.51)	103.5 (38.09, 281.23)	6.62 (5.32)	98.67 (42.6)
general disorders and administration site conditions	thirst	473	75.79 (69.08, 83.15)	74.17 (67.25, 81.81)	6.16 (6.03)	71.67 (66.33)
general disorders and administration site conditions	haemorrhagic cyst	10	72.3 (38.49, 135.83)	72.27 (38.6, 135.32)	6.13 (5.26)	69.89 (41.24)
investigations	urine osmolarity increased	3	67.62 (21.41, 213.5)	67.61 (21.27, 214.9)	6.03 (4.59)	65.53 (25.04)
investigations	blood urea nitrogen/creatinine ratio decreased	3	65.5 (20.76, 206.71)	65.5 (20.61, 208.19)	5.99 (4.55)	63.54 (24.29)
hepatobiliary disorders	portal vein occlusion	3	64.17 (20.34, 202.42)	64.16 (20.19, 203.93)	5.96 (4.52)	62.28 (23.82)
investigations	blood sodium abnormal	21	57.44 (37.22, 88.62)	57.38 (37.28, 88.31)	5.8 (5.19)	55.88 (38.87)
general disorders and administration site conditions	cyst rupture	14	56.14 (33.01, 95.47)	56.1 (33.05, 95.23)	5.77 (5.03)	54.67 (35.06)
investigations	alanine aminotransferase decreased	12	52.42 (29.56, 92.98)	52.4 (29.68, 92.51)	5.68 (4.88)	51.14 (31.66)
investigations	blood sodium decreased	309	50.43 (45.02, 56.5)	49.74 (44.22, 55.95)	5.6 (5.44)	48.61 (44.2)
investigations	blood urea increased	243	48.38 (42.57, 54.98)	47.85 (42.54, 53.82)	5.55 (5.36)	46.81 (42.06)
renal and urinary disorders	nocturia	199	47.12 (40.92, 54.26)	46.7 (40.71, 53.57)	5.51 (5.31)	45.7 (40.61)
investigations	urine albumin/creatinine ratio increased	7	45.58 (21.55, 96.38)	45.56 (21.63, 95.95)	5.48 (4.47)	44.61 (23.84)
neoplasms benign, malignant and unspecified (incl cysts and polyps)	liver carcinoma ruptured	4	45.57 (16.92, 122.72)	45.56 (16.77, 123.8)	5.48 (4.2)	44.61 (19.47)
investigations	blood chloride increased	22	44.55 (29.2, 67.97)	44.51 (28.92, 68.51)	5.45 (4.85)	43.6 (30.62)
investigations	blood osmolarity increased	4	43.22 (16.06, 116.33)	43.21 (15.9, 117.41)	5.4 (4.12)	42.36 (18.5)
neoplasms benign, malignant and unspecified (incl cysts and polyps)	metastatic bronchial carcinoma	3	41.64 (13.28, 130.59)	41.64 (13.36, 129.78)	5.35 (3.92)	40.85 (15.7)
investigations	glomerular filtration rate decreased	180	41.46 (35.75, 48.08)	41.13 (35.86, 47.18)	5.33 (5.12)	40.36 (35.65)
neoplasms benign, malignant and unspecified (incl cysts and polyps)	bronchial carcinoma	12	39.63 (22.38, 70.16)	39.61 (22.44, 69.93)	5.28 (4.49)	38.89 (24.11)
neoplasms benign, malignant and unspecified (incl cysts and polyps)	small cell lung cancer metastatic	5	39.55 (16.33, 95.82)	39.54 (16.37, 95.52)	5.28 (4.11)	38.83 (18.52)
investigations	aspartate aminotransferase decreased	7	38.52 (18.24, 81.36)	38.51 (18.29, 81.1)	5.24 (4.23)	37.83 (20.24)
investigations	urine output increased	38	37.56 (27.24, 51.78)	37.5 (27.41, 51.31)	5.2 (4.75)	36.85 (28.17)
metabolism and nutrition disorders	hyperuricaemia	53	34.91 (26.61, 45.82)	34.83 (26.47, 45.83)	5.1 (4.71)	34.28 (27.31)
investigations	carbon dioxide decreased	12	34.85 (19.7, 61.67)	34.83 (19.73, 61.49)	5.1 (4.31)	34.28 (21.27)
hepatobiliary disorders	hepatorenal syndrome	19	32.3 (20.53, 50.82)	32.27 (20.56, 50.65)	4.99 (4.35)	31.8 (21.76)
nervous system disorders	hepatic encephalopathy	102	29.55 (24.29, 35.94)	29.41 (24.18, 35.78)	4.86 (4.58)	29.02 (24.63)
renal and urinary disorders	kidney enlargement	11	28.8 (15.88, 52.22)	28.78 (15.99, 51.82)	4.83 (4.01)	28.41 (17.26)
injury, poisoning and procedural complications	drug titration error	25	27.36 (18.44, 40.61)	27.33 (18.47, 40.45)	4.75 (4.2)	26.99 (19.4)
investigations	anion gap decreased	3	27.34 (8.75, 85.41)	27.34 (8.77, 85.21)	4.75 (3.33)	27 (10.41)
investigations	urine protein/creatinine ratio increased	7	25.66 (12.17, 54.07)	25.65 (12.18, 54.02)	4.66 (3.66)	25.35 (13.59)
neoplasms benign, malignant and unspecified (incl cysts and polyps)	small cell carcinoma	3	24.66 (7.9, 76.98)	24.66 (7.91, 76.86)	4.61 (3.18)	24.38 (9.41)
gastrointestinal disorders	oesophageal varices haemorrhage	19	23.64 (15.04, 37.16)	23.62 (15.05, 37.07)	4.55 (3.91)	23.37 (16)
investigations	blood uric acid increased	48	23.31 (17.54, 31)	23.27 (17.69, 30.62)	4.52 (4.12)	23.02 (18.14)
investigations	blood phosphorus abnormal	4	23.16 (8.65, 62.06)	23.16 (8.69, 61.71)	4.52 (3.24)	22.92 (10.05)
hepatobiliary disorders	hepatic cyst	28	23.02 (15.86, 33.42)	23 (15.85, 33.38)	4.51 (3.98)	22.76 (16.66)
neoplasms benign, malignant and unspecified (incl cysts and polyps)	small cell lung cancer	12	22.33 (12.64, 39.44)	22.32 (12.64, 39.4)	4.47 (3.68)	22.09 (13.72)
infections and infestations	infected cyst	10	21.57 (11.57, 40.23)	21.56 (11.51, 40.37)	4.42 (3.56)	21.35 (12.68)
investigations	blood chloride decreased	15	21.3 (12.81, 35.43)	21.28 (12.78, 35.42)	4.4 (3.69)	21.08 (13.77)

ROR, reporting odds ratio; CI, confidence interval; PRR, proportional reporting ratio; IC, information component; IC 025, the lower limit of 95% CI of the IC; EBGM, empirical Bayesian geometric mean; EBGM 05: the lower limit of 95% CI of EBGM.

### 3.3 Contaminant drugs frequency of tolvaptan

In the analysis of 7,486 reports of suspected adverse drug reactions related to tolvaptan, the most commonly contaminant drugs were furosemide (1,512 cases), followed by spironolactone (697 cases) and amlodipine (601 cases). The drugs ranked fourth and fifth were aspirin (403 cases) and carvedilol (363 cases). When patients are prescribed these medications, healthcare providers should adjust dosages based on individual circumstances and perform appropriate monitoring to minimize the occurrence of adverse reactions. As shown in Supplementary Figure S2.

### 3.4 Subgroup analysis

Subsequently, we performed subgroup analyses to mitigate the potential confounding effects of demographic characteristics on the results. In the <18 years subgroup, the highest number of cases was associated with “product use issues”; in the 18–65 years subgroup, “thirst” was the most reported adverse event; and in the ≥65 years subgroup, “hypernatremia” was the most common. Further analysis of the top five ADEs in each subgroup revealed that wrong technique in product usage process, seizures, and product administered to patient of inappropriate age were reported only in the <18 years subgroup. Polyuria, alanine aminotransferase increased, and nocturia were only reported in the 18–65 years subgroup, while cardiac failure and product use in unapproved indication were more common in patients aged ≥65 years. As shown in Supplementary Figure S3. Additionally, we assessed the differences in ADEs across subgroups by weight (Supplementary Figure S4) and gender (Supplementary Figure S5). These subgroup analyses provide a method for comparing signal values between different groups, enabling the identification of similarities and differences. This information is critical for more detailed clinical management, and guiding clinicians to adjust treatment based on the characteristics of specific subgroups is essential.

## 4 Discussion

Tolvaptan is widely used in the treatment of hyponatremia, heart failure, and polycystic kidney disease. A prospective observational study conducted in seven European countries included 252 hospitalized patients with hyponatremia caused by SIADH. The results showed that tolvaptan effectively increased serum sodium levels in patients with SIADH-related hyponatremia in clinical practice, with a favorable safety profile. However, some patients experienced side effects such as nausea, urinary tract infections, constipation, and dry mouth. The study also emphasized the importance of monitoring serum sodium levels during treatment to prevent the risk of rapid correction of hyponatremia (Estilo et al., 2021). A retrospective study previously reported that tolvaptan rapidly increased urine output, reduced body weight, improved hyponatremia, and alleviated congestion symptoms. However, long-term trials showed that tolvaptan did not significantly reduce rehospitalization rates or mortality in heart failure patients. The overall tolerability of tolvaptan was good, with common side effects including dry mouth and thirst. In rare cases, electrolyte imbalances such as hypokalemia and hypernatremia occurred but with low incidence rates (Ambrosy et al., 2011). In a phase 3, multicenter, double-blind, randomized controlled trial, patients with ADPKD who were treated with tolvaptan over a 3-year period showed significantly lower rates of cyst growth, decline in kidney function, and incidence of back pain compared to the placebo group. However, AEs such as thirst and polyuria were reported (Torres et al., 2012). To date, there are few large-scale real-world studies on tolvaptan. Previous studies using the FAERS database have focused on specific complications associated with tolvaptan, such as thromboembolic events and severe liver disease (Aiello et al., 2021; Uno et al., 2024). Based on real-world large-sample data, we have collected and assessed the pharmacovigilance of tolvaptan after its market approval. Our goal is to analyze new and meaningful adverse reactions, providing guidance for clinical use and alerting clinicians to potential complications.

This study observed that the majority of ADR reports involved patients aged 45 and above, with only 11.1% of reports coming from patients under 45. This is likely due to the fact that tolvaptan is approved for treating clinically significant hypervolemic and euvolemic hyponatremia, including in patients with heart failure and cirrhosis, conditions more commonly seen in elderly individuals with underlying diseases. Most of the ADR reports (43.55%) were submitted by healthcare professionals, indicating that tolvaptan is primarily prescribed by clinicians who also regularly monitor patients during treatment. Regarding the countries reporting ADRs, the United States accounted for the largest proportion (41.77%), possibly due to earlier drug approval and higher prescription rates in that country. The high proportion of serious reports involving death, hospitalization, and life-threatening events highlights the importance of healthcare professionals remaining vigilant when treating patients with this drug. In the event of severe ADRs, prompt management is essential. In our study, Supplementary Figure S1 highlights the top 10 signals associated with hospitalization and mortality in patients receiving tolvaptan. Understanding these signals is crucial for improving clinical management and optimizing patient safety. The three signals most frequently associated with mortality were cardiac failure (144 occurrences), product use in unapproved indications (118 occurrences), and hypernatremia (110 occurrences). For hospitalization, the leading signals were product use in unapproved indications (151 occurrences), wrong technique in product usage (147 occurrences), and dehydration (146 occurrences). The association between heart failure and mortality signals is particularly concerning. Previous studies have shown that heart failure patients are particularly vulnerable to electrolyte imbalances and fluid shifts, which can lead to worsened cardiac function and increased mortality risk (Yancy et al., 2017). The frequent occurrence of heart failure as a signal suggests that careful monitoring of cardiac status is crucial when administering tolvaptan, especially in patients with pre-existing cardiac conditions. The high frequency of signals related to the use of tolvaptan in unapproved indications raises significant concerns about off-label prescribing. Off-label use of drugs, while common, can lead to unintended consequences, as the safety profile of the drug may not be well-established for these conditions. It is important to emphasize that the risks associated with off-label use may not be adequately captured in clinical trials, and practitioners should exercise caution when prescribing tolvaptan for indications outside of approved guidelines. The high incidence of this signal suggests that further research is needed to clarify the risk-benefit ratio of using tolvaptan in unapproved settings. Hypernatremia is another critical signal associated with mortality in our analysis. Tolvaptan, as a vasopressin antagonist, can affect water and sodium balance, and hypernatremia is a recognized risk associated with its use. Severe hypernatremia can lead to serious neurological complications, including seizures, coma, and death. This finding underscores the need for careful monitoring of sodium levels in patients receiving tolvaptan, particularly in those with renal or endocrine dysfunction who may be at increased risk. The signal regarding wrong technique in product usage also warrants attention, as improper administration of tolvaptan could lead to suboptimal therapeutic outcomes and increase the risk of AEs. Patient education on the correct usage of the drug is vital to prevent such occurrences. This finding emphasizes the need for better communication and training for both healthcare providers and patients regarding the proper use of tolvaptan, especially considering its complex dosing regimen and potential side effects. 

Disproportionality analysis identified significant AEs associated with tolvaptan across various SOCs, including hepatobiliary disorders, renal and urinary disorders, and metabolic and nutritional disorders. One study on ADPKD patients using tolvaptan found that the drug can cause irreversible and fatal liver injury (Woodhead et al., 2017). The mechanism by which tolvaptan induces liver damage remains unclear. A study by Slizgi et al. suggested that inhibition of hepatic bile acid transport may be one of the biological mechanisms contributing to tolvaptan-related liver injury in ADPKD patients (Slizgi et al., 2016). In addition, studies have shown that the stress of drugs on liver cells leads to the production of neoantigens, induces the attack of the adaptive immune system on liver cells, the destruction of liver cell transporters, and the damage of bile salt excretion pumps can all lead to liver cell damage, which is the result of various metabolic polymorphisms (Alpers et al., 2023). There is ongoing debate about whether tolvaptan affects renal function. In a prospective, double-blind, placebo-controlled trial involving patients with congestive heart failure (Felker et al., 2017), patients treated with tolvaptan experienced a higher incidence of renal function deterioration within 72 h compared to the placebo group. However, these changes were transient, and by the 72-h mark, renal function in both groups was similar. The effect of tolvaptan on renal function varies across different patient populations, necessitating further research. For patients on long-term tolvaptan therapy, close monitoring of renal function markers such as serum creatinine and uric acid is essential. Hypernatremia and dehydration are common adverse reactions during tolvaptan use, underscoring the need for careful monitoring of these parameters during treatment.

At the PT level, this study identified positive signals of specific adverse reactions such as thirst, hypernatremia, dehydration, and polyuria, all of which are mentioned in the drug label, thereby enhancing the reliability of our findings. Additionally, our study uncovered AEs not documented in the drug label, such as renal cyst rupture, renal cyst infection, polycystic liver disease, and hemorrhage of renal cysts. These adverse reactions exhibited strong signal intensity and warrant special attention. The potential reasons for these findings could be attributed to the fact that tolvaptan is approved for the treatment of patients with ADPKD. As ADPKD progresses, cysts may increase in size and number, leading to renal dysfunction and complications such as hepatic cysts or cysts in other organs. Most ADPKD patients eventually develop end-stage renal disease and require renal replacement therapy (Blair, 2019). Given the strong association between these signals and the progression of ADPKD itself, we recommend closely monitoring the progression of the primary disease in patients undergoing tolvaptan treatment for ADPKD. Should any of the above ADEs occur, timely identification and intervention are crucial to ensure patient safety.

A decrease in urine osmolality, although a rare ADE not mentioned in the drug label, showed a strong signal, which may also be related to the underlying disease. Previous studies have reported that patients with ADPKD may develop a defect in urine concentration even before the decline in glomerular filtration rate (GFR) occurs (Gabow et al., 1989). The severity of this impairment correlates with the number and size of cysts as well as the remaining volume of normal renal parenchyma; the more severe the structural abnormalities, the greater the impairment in the kidney’s ability to concentrate urine. A prior multicenter, prospective analysis suggested a trend toward a greater reduction in urine sodium and osmolality in patients receiving tolvaptan treatment (Gheorghiade et al., 2006). One proposed mechanism is that cyst formation predominantly occurs in the collecting ducts at the corticomedullary junction (D’Angelo et al., 1975). Consequently, as the number of renal cysts increases, damage to normal renal tissue worsens, leading to further deterioration of renal function and a decline in the kidney’s concentrating ability. Therefore, during tolvaptan treatment for ADPKD, a decrease in urine osmolality may indicate disease progression. This parameter could potentially aid in disease monitoring, drug adjustment, and prognosis evaluation.

In this study, the incidence of osmotic demyelination syndrome (ODS) was relatively high, and the ADE signal was strong. ODS is characterized by symptoms such as dysarthria, mutism, dysphagia, drowsiness, emotional changes, seizures, coma, and even death. The black box warning in the drug label for tolvaptan indicates that patients must take the medication in a hospital setting where serum sodium levels can be closely monitored. Rapid increases in serum sodium concentrations can lead to the development of severe ODS (Gabow et al., 1989). Multiple previous studies have confirmed that rapid correction of hyponatremia increases the incidence of ODS, and this complication should be carefully monitored during treatment (Ho et al., 2019; Arecco et al., 2024; Krisanapan et al., 2023). The primary mechanism involves abrupt changes in osmotic pressure inside and outside the cells, leading to damage to brain cells, particularly oligodendrocytes. When the myelin structure is damaged, nerve conduction is impaired, resulting in neurological symptoms, including abnormalities in motor function, consciousness, behavior, and cognition. Therefore, in patients with hyponatremia, particularly those with severe malnutrition, alcoholism, or advanced liver disease, a slower rate of sodium correction is recommended to mitigate the risk of ODS.

It is noteworthy that the ADE signal related to benign, malignant, and neoplasms of uncertain behavior was particularly strong, especially in lung-related tumors, such as metastatic bronchial carcinoma, bronchial carcinoma, metastatic small cell lung cancer, and small cell lung carcinoma. Currently, there is no evidence suggesting that tolvaptan induces lung tumors or exacerbates tumor progression. On the contrary, some studies have shown that tolvaptan may have an inhibitory effect on tumor proliferation. In an *in vitro* study by Naldi et al., tolvaptan was found to inhibit tumor growth in a xenograft model of small cell lung cancer in mice (Naldi et al., 2024). A case report evaluating the efficacy and safety of long-term tolvaptan use in patients with small cell lung cancer and SIADH showed that the main symptoms reported were fatigue, stomach pain, and mild coughing, which were likely related to the underlying disease and chemotherapy. No serious AEs were observed during a treatment period of up to 1 year. Hyponatremia is common in cancer patients, primarily due to SIADH caused by ectopic secretion of antidiuretic hormone (Bordi et al., 2015). The most common cause of hyponatremia in cancer patients is SIADH, which may result from tumors secreting arginine vasopressin, and it is most frequently observed in lung cancer (Marroncini et al., 2021). Approximately 15% of small cell lung cancer cases are associated with SIADH (Bordi et al., 2015; Ren and Yang, 2021). Many patients present with persistent hyponatremia even before tumor diagnosis, prompting the use of tolvaptan for correcting hyponatremia. Given the complexity of the disease, medication regimens, and various treatment plans in cancer patients, it can be challenging to distinguish whether clinical symptoms are due to ADEs or disease progression. Therefore, the safety of tolvaptan use in cancer patients warrants more systematic evaluation.

Sexual dimorphism has been shown to influence the pharmacokinetics of drugs, including absorption, distribution, metabolism, and excretion, leading to differences in adverse drug reactions (ADRs) between males and females (Farkouh et al., 2020). However, there is currently a lack of gender-specific ADR reports related to tolvaptan therapy. To further investigate the relationship between gender and drug-induced AEs, we conducted a gender-based subgroup analysis. As shown in Supplementary Figure S5, women are more prone to AEs such as thirst, product use in unapproved indication, inappropriate schedule of product administration, wrong technique in product usage process, and blood sodium decreased. In contrast, men, in addition to thirst, are more likely to experience hypernatremia, renal impairment, blood creatinine increased, and wrong technique in product usage process. A better understanding of gender-related ADRs can help improve the safety and efficacy of medications for both men and women, thereby optimizing therapeutic strategies. Future clinical trials and mechanistic studies are needed to validate and elucidate these gender-specific ADRs, ultimately providing more personalized treatment approaches for both genders.

Although this study provides reliable scientific evidence for the safety assessment of tolvaptan from multiple perspectives, certain limitations remain: (1) The data in this study were sourced from the FARES spontaneous reporting system, which, despite its large database and wide population coverage, may lead to reporting bias and incomplete information; The demographic data of patients included in the FAERS database may not fully represent the broader population using tolvaptan. This may limit the generalizability of the study findings; (2) Since the number of patients using tolvaptan without experiencing ADEs could not be obtained, it was not possible to calculate the overall incidence of ADEs; (3) Countries and regions with a higher number of reports may introduce sampling bias. To gain a more comprehensive and accurate understanding, future studies could be combined with basic research to explore the biological basis of newly identified AEs, such as those associated with lung tumors and kidney complications found in this study. Randomized controlled trials were further validated through clinical study design to clarify the causal relationship between tolvaptan and identified AEs to better assess the safety risk of tolvaptan. (4) Furthermore, the dose-response relationship between the drug and AEs can provide valuable insights into the drug’s risk-benefit profile. However, the analysis is hindered by a large amount of missing data, which obstructs meaningful dose-response assessments.

## 5 Conclusion

This study conducted a mining analysis of the ADE reports related to tolvaptan in the FARES database, providing a solid scientific foundation for the safety assessment of tolvaptan through multi-angle and multi-level analysis. The results suggest that, in clinical practice, special attention should be paid to the high incidence and strong signals of ADEs caused by tolvaptan, including systemic disorders and reactions at the administration site (thirst), renal and urinary disorders (renal impairment, polyuria), metabolic and nutritional disorders (hypernatremia, dehydration), and hepatobiliary disorders (abnormal liver function). Notably, while some adverse reactions, such as decreased urine osmolality and osmotic demyelination syndrome, occur relatively infrequently, their strong signal intensity warrants further attention and research. The study also uncovered several unique ADEs not explicitly mentioned in the drug label, such as renal cyst rupture, renal cyst infection, polycystic liver disease, hemorrhage of renal cysts, and lung-related tumors, including metastatic bronchial carcinoma, bronchial carcinoma, metastatic small cell lung cancer, and small cell lung carcinoma. These findings suggest that healthcare professionals should exercise greater caution when prescribing tolvaptan, and patients should be made aware of these potential adverse reactions. Before initiating treatment with tolvaptan, a thorough evaluation should be conducted, and patients should be closely monitored during treatment for these ADEs and the progression of their underlying disease. If ADEs or disease progression occur, timely intervention is necessary to ensure the safety of the patient.

## Data Availability

The raw data supporting the conclusions of this article will be made available by the authors, without undue reservation.
