# Supplementary material for: Safety assessment of tolvaptan: real-world adverse event analysis using the FAERS database
Source: Front Pharmacol. 2025 Jan 9;15:1509310. doi: 10.3389/fphar.2024.1509310 (PMC11754202; doi:10.3389/fphar.2024.1509310)

**Table S1. Frequency Distribution of Tolvaptan Dosing Schedules.**

| Dosage and frequency of drug use | n |
| --- | --- |
| 0.05 mg/kg, qd | 2 |
| 0.1 mg/kg, single administration | 1 |
| 0.14 mg/kg, qd | 1 |
| 0.15 mg/kg, qd | 1 |
| 0.15 mg/kg, single administration | 1 |
| 0.28 mg/kg, qd | 1 |
| 0.5 mg/kg, qd | 1 |
| 0.5-1 mg/kg, qd | 1 |
| 0.9 mg/kg, daily dose | 1 |
| 1 mg, qd | 2 |
| 1.1 mg, qd | 1 |
| 1.625 mg, qd | 1 |
| 1.875 mg, qd | 3 |
| 10 mg, qd | 1 |
| 105 mg, qd | 1 |
| 11.25 mg, qd | 6 |
| 120 mg, daily dose | 23 |
| 120 mg, qd | 19 |
| 135 mg, qd | 1 |
| 15 mg, 4/W | 1 |
| 15 mg, 5/W | 1 |
| 15 mg, as necessary | 6 |
| 15 mg, bid | 34 |
| 15 mg, biw | 29 |
| 15 mg, mwf; 7.5 mg stths | 1 |
| 15 mg, Q10D | 1 |
| 15 mg, Q3D | 14 |
| 15 mg, Q4D | 2 |
| 15 mg, Q5D | 2 |
| 15 mg, qd | 1029 |
| 15 mg, qid | 2 |
| 15 mg, qiw | 4 |
| 15 mg, qod | 59 |
| 15 mg, qod to Q3D | 1 |
| 15 mg, qw | 19 |
| 15 mg, single administration | 35 |
| 15 mg, tid | 3 |
| 15 mg, tiw | 39 |
| 16 mg, qd | 8 |
| 18.75 mg, daily dose | 1 |
| 18.75 mg, qd | 1 |
| 180 mg, qd | 1 |
| 2 mg, qd | 1 |
| 2.5 mg, qd | 1 |
| 20 or 40 mg, qd | 1 |
| 22.5 mg, bid | 5 |
| 22.5 mg, daily dose | 4 |
| 22.5 mg, qd | 5 |
| 3.25 mg, qd | 3 |
| 3.5 mg, bid | 1 |
| 3.5 mg, qd | 1 |
| 3.75 mg, bid | 1 |
| 3.75 mg, qd | 318 |
| 3.75 mg, qod | 4 |
| 3.75 mg, tiw | 1 |
| 3.75 mg, biw | 1 |
| 3.75 or 7.5 mg, qd | 2 |
| 3.75 to 15 mg, qd | 1 |
| 3.8 mg, qd | 3 |
| 30 mg, bid | 20 |
| 30 mg, biw | 2 |
| 30 mg, daily dose | 11 |
| 30 mg, qd | 284 |
| 30 mg, qid | 1 |
| 30 mg, qiw | 1 |
| 30 mg, qod | 4 |
| 30 mg, single administration | 7 |
| 30 mg, tiw | 3 |
| 37.5 mg, daily dose | 2 |
| 4.2 mg, qd | 1 |
| 40 mg, qd | 1 |
| 40 mg,daily dose | 1 |
| 45 mg, bid | 2 |
| 45 mg, daily dose | 7 |
| 45 mg, qd | 580 |
| 5 mg, bid | 1 |
| 5 mg, qd | 2 |
| 5 to 10 mg, qd | 1 |
| 50 mg, daily dose | 2 |
| 52.5 mg, daily dose | 1 |
| 60 mg, 1/month | 1 |
| 60 mg, bid | 3 |
| 60 mg, daily dose | 87 |
| 60 mg, qd | 217 |
| 67.5 mg, daily dose | 2 |
| 7 mg, Q3D to Q4D | 1 |
| 7 mg,qd | 1 |
| 7.5 mg to 15 mg, qd | 14 |
| 7.5 mg, 4-5x/month | 1 |
| 7.5 mg, 5/W | 1 |
| 7.5 mg, as necessary | 3 |
| 7.5 mg, bid | 9 |
| 7.5 mg, biw | 12 |
| 7.5 mg, biw to tiw | 1 |
| 7.5 mg, Q3D | 4 |
| 7.5 mg, qd | 822 |
| 7.5 mg, qid | 1 |
| 7.5 mg, qiw | 1 |
| 7.5 mg, qod | 35 |
| 7.5 mg, qw | 3 |
| 7.5 mg, single administration | 3 |
| 7.5 mg, tid | 1 |
| 7.5 mg, tiw | 21 |
| 75 mg, daily dose | 1 |
| 75 mg, qd | 5 |
| 8 mg, bid | 2 |
| 8 mg, qd | 25 |
| 90 mg, daily dose | 24 |
| 90 mg, qd | 200 |
| day 1: 15mg, qd: day 2-3: 7.5mg, qd | 1 |
| Unknown/Undetermined | 3336 |

4/W, 4 times per week; 5/W, 5 times per week; bid, twice a day; biw, twice a week; mwf, Monday/Wednesday/Friday; Q10D, every 10 days; Q3D, every 3 days; Q4D, every 4 days; Q5D, every 5 days; qd, once a day; qid, four times a day; qiw, four times a week; qod, every other day; qw, once a week; stths, Saturday/Thursday; tid, three times a day; tiw, three times a week.

**Figure S1. Preferred Terms (PTs) Associated with Death and Hospitalization: (A) Top 10 PTs Associated with Death; (B) Top 10 PTs Associated with Hospitalization.**


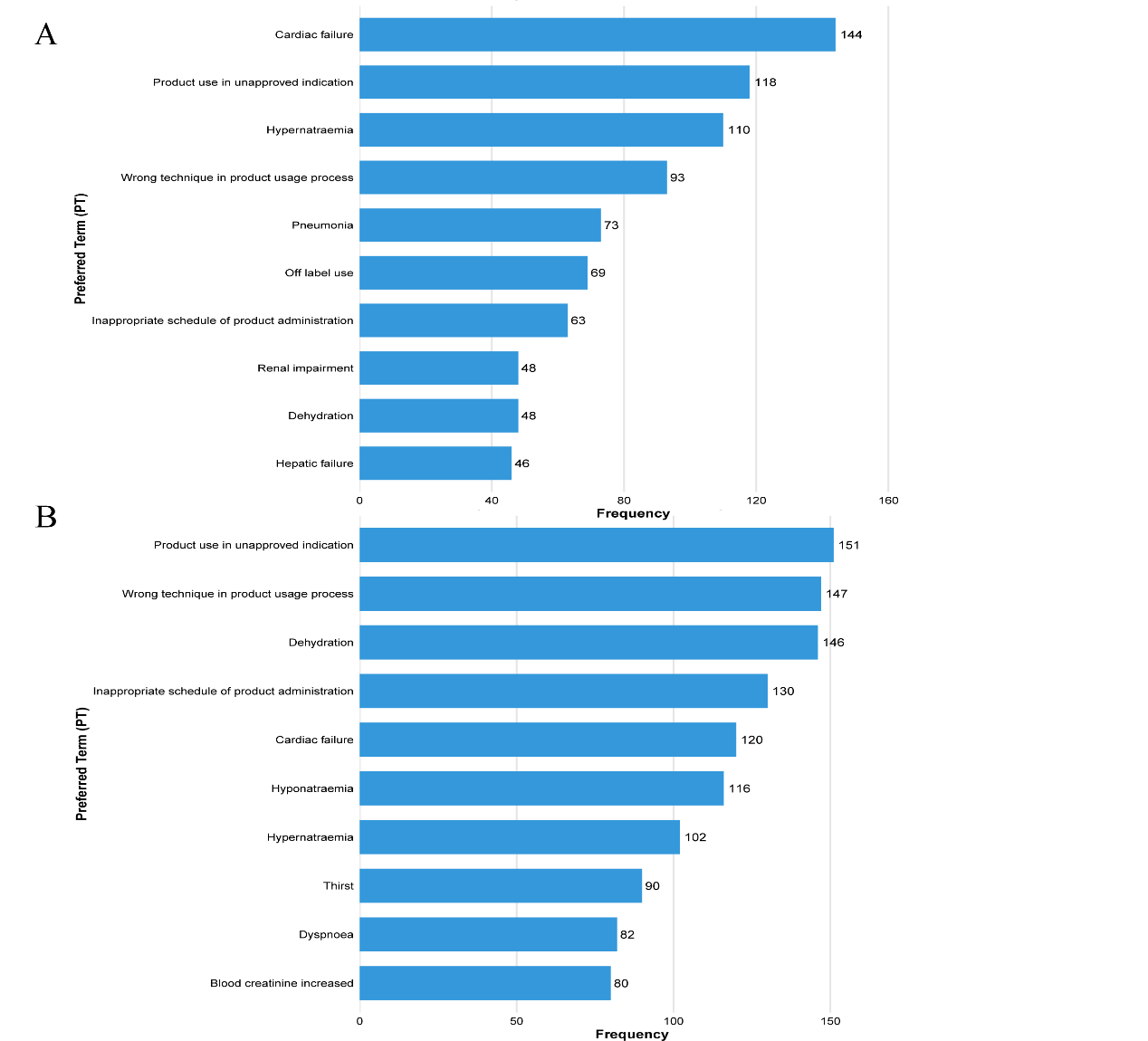


**Figure S2. Frequency of Contaminant Drugs Associated with Tolvaptan.**


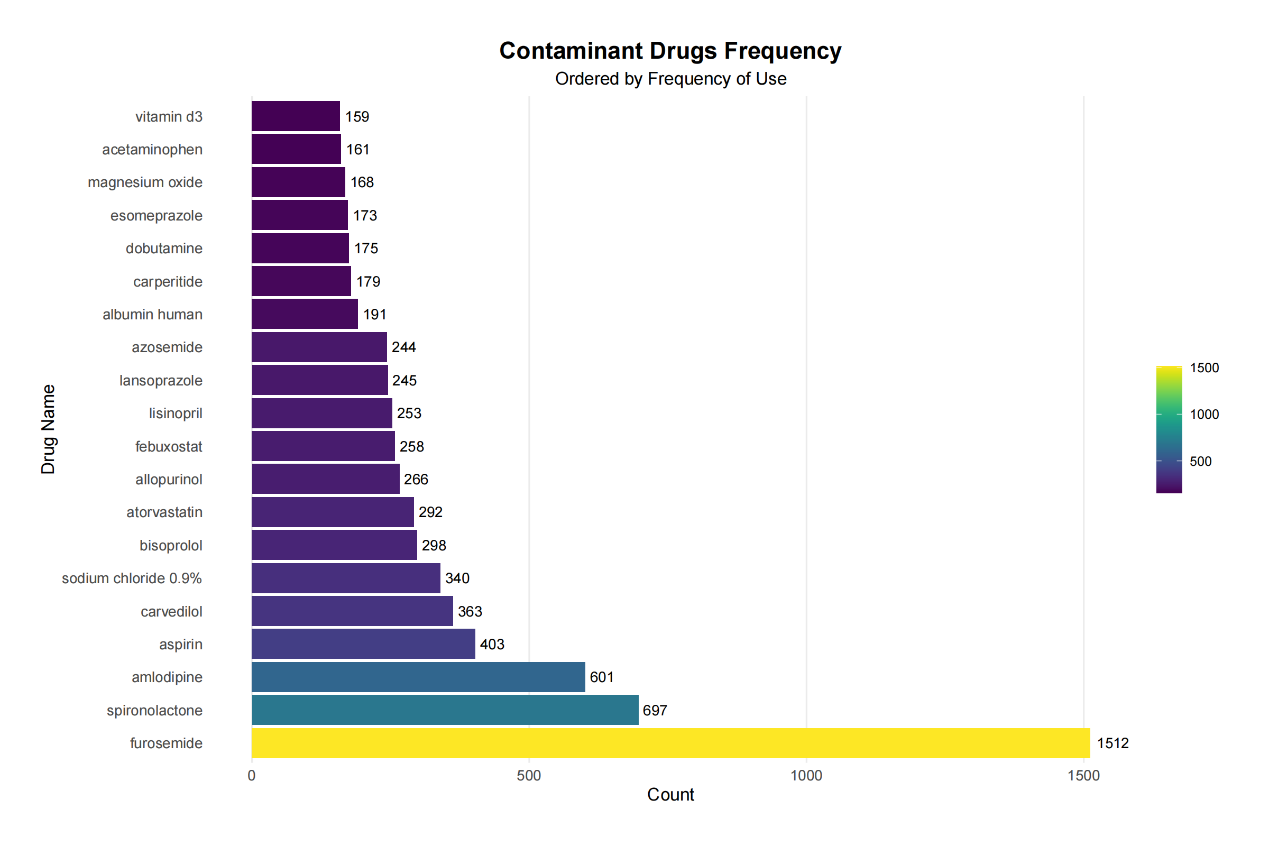


**Figure S3. Age-Based Subgroup Analysis of Tolvaptan-Related Adverse Drug Events (ADEs): (A) Forest Plot of the Top 5 Events by Case Numbers (Age 18–65); (B) Forest Plot of the Top 5 Events by Case Numbers (Age ≥ 65); (C) Forest Plot of the Top 5 Events by Case Numbers (Age < 18).**


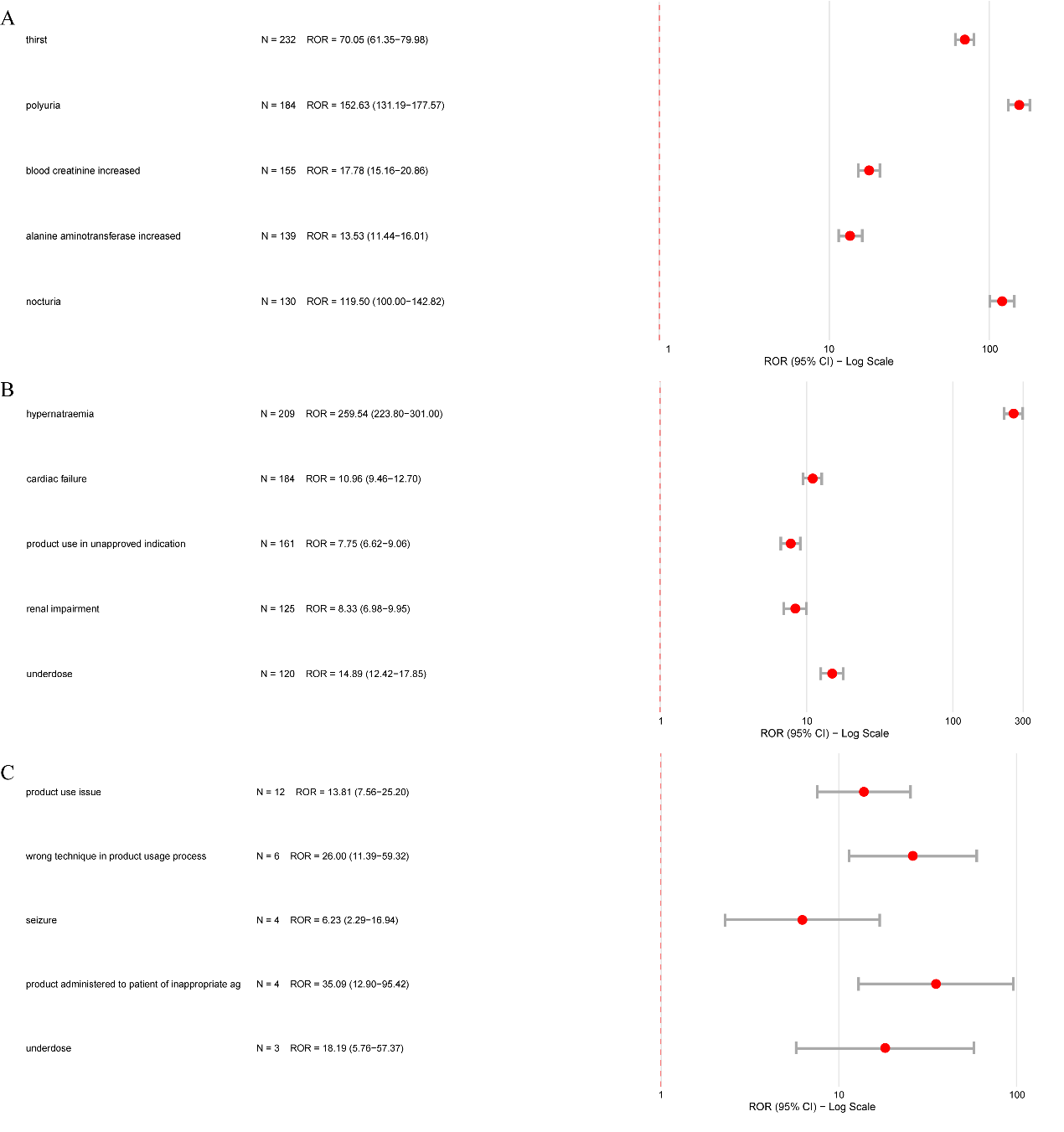


**Figure S4. Weight-Based Subgroup Analysis of Tolvaptan-Related Adverse Drug Events (ADEs): (A) Forest Plot of the Top 5 Events by Case Numbers (60–100 kg); (B) Forest Plot of the Top 5 Events by Case Numbers (≥100 kg); (C) Forest Plot of the Top 5 Events by Case Numbers (<60 kg).**


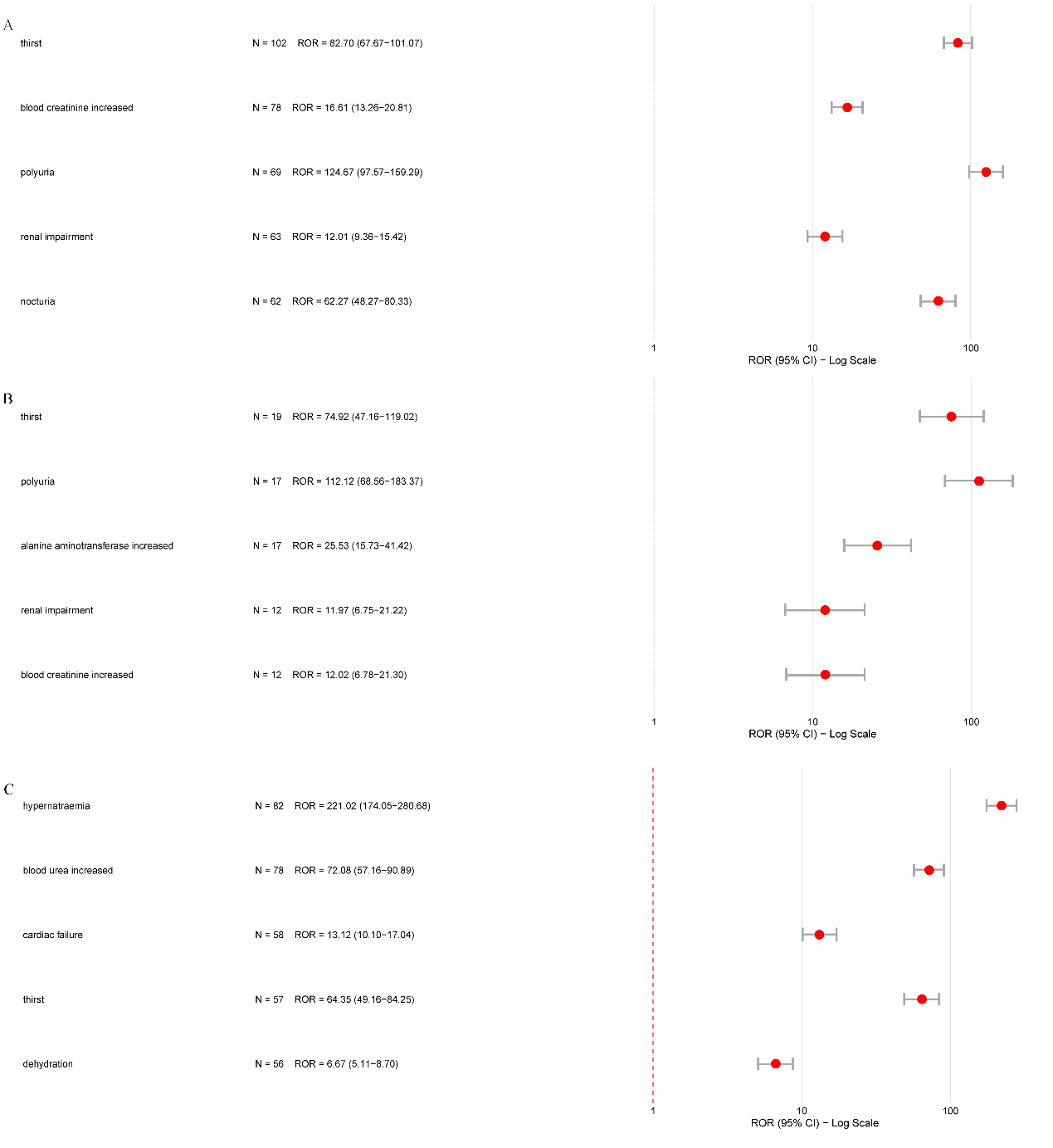


**Figure S5. Gender-Based Subgroup Analysis of Tolvaptan-Related Adverse Drug Events (ADEs): (A) Forest Plot of the Top 5 Events by Case Numbers (Female); (B) Forest Plot of the Top 5 Events by Case Numbers (Male).**


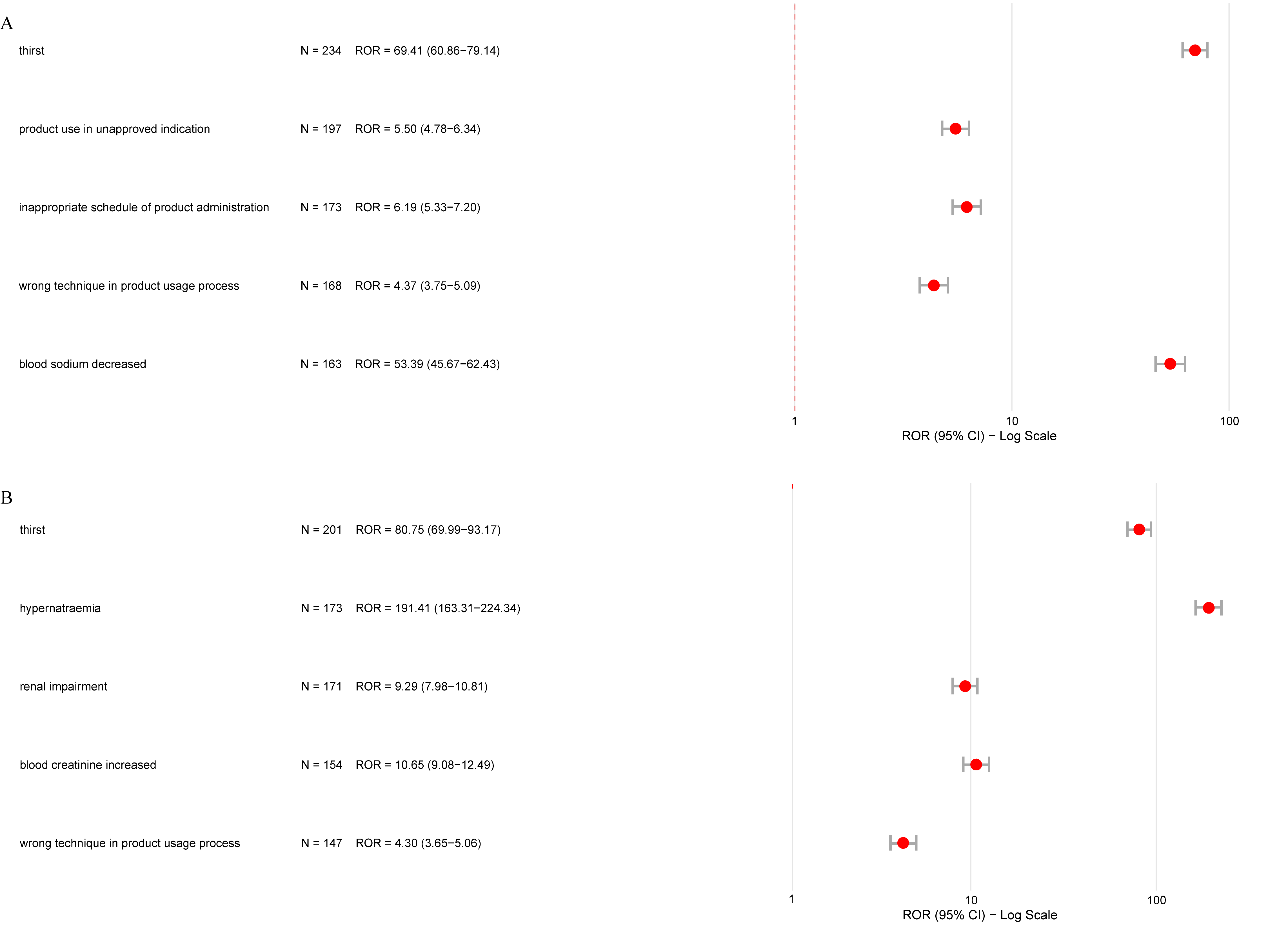

Supplement: Supplementary file 1 [file DataSheet1.docx]
